# An Enhanced Gas Sensor Data Classification Method Using Principal Component Analysis and Synthetic Minority Over-Sampling Technique Algorithms

**DOI:** 10.3390/mi15121501

**Published:** 2024-12-16

**Authors:** Xianzhang Zeng, Muhammad Shahzeb, Xin Cheng, Qiang Shen, Hongyang Xiao, Cao Xia, Yuanlin Xia, Yubo Huang, Jingfei Xu, Zhuqing Wang

**Affiliations:** 1 School of Mechanical Engineering, Sichuan University, Chengdu 610065, China; zengxianzhang@scu.edu.cn (X.Z.); muhammadshahzeb133@gmail.com (M.S.); shenqiang@stu.scu.edu.cn (Q.S.); xiacao_30@scu.edu.cn (C.X.); yuanlin.xia@scu.edu.cn (Y.X.); huangyubo@scu.edu.cn (Y.H.); 2 Graduate School of International Cultural Studies, Tohoku University, Kawauchi 41, Aoba Ku, Sendai 980-8577, Miyagi, Japan; cheng.xin.t2@dc.tohoku.ac.jp; 3 Pittsburgh Institute, Sichuan University, Chengdu 610225, China; 13990184114@163.com; 4 Rehabilitation Medicine Center, West China Hospital, Sichuan University, Chengdu 610041, China; 5 Key Laboratory of Rehabilitation Medicine in Sichuan Province, West China Hospital, Sichuan University, Chengdu 610041, China

**Keywords:** machine learning, gas sensor, PCA, SMOTE, KNN

## Abstract

This study addresses the challenge of multi-dimensional and small gas sensor data classification using a gelatin–carbon black (CB-GE) composite film sensor, achieving 91.7% accuracy in differentiating gas types (ethanol, acetone, and air). Key techniques include Principal Component Analysis (PCA) for dimensionality reduction, the Synthetic Minority Over-sampling Technique (SMOTE) for data augmentation, and the Support Vector Machine (SVM) and K-Nearest Neighbor (KNN) algorithms for classification. PCA improved KNN and SVM classification, boosting the Area Under the Curve (AUC) scores by 15.7% and 25.2%, respectively. SMOTE increased KNN’s accuracy by 2.1%, preserving data structure better than polynomial fitting. The results demonstrate a scalable approach to enhancing classification accuracy under data constraints. This approach shows promise for expanding gas sensor applicability in fields where data limitations previously restricted reliability and effectiveness.

## 1. Introduction

In human–computer interaction, gas sensors are crucial, serving as the sensory organs of robots [1,2]. In a previous work, our group developed a highly sensitive and selective gas sensor using a novel gelatin–carbon black (CB-GE) composite film, fabricated via the thin-film needle coating (TFNC) method on a microelectromechanical system (MEMS) platform [3]. This sensor demonstrated strong sensitivity and reliable response across varying humidity levels and specific gasses, such as ethanol and acetone, with a fast response time and high reproducibility. The CB-GE film allowed for effective gas detection by leveraging changes in electrical resistance as it absorbed analyte molecules. Building on this foundation, we now address the complexities in interpreting the multi-dimensional data generated by such gas sensors. Due to the intricate patterns and limited data within the sensor’s signal output, we propose integrating machine learning algorithms to classify these signals effectively.

Machine learning algorithms are widely used to analyze sensor data, enabling robots to engage with their environments more actively [4,5]. Machine learning-based signal processing has gained significant importance [6,7,8,9], but classification accuracy heavily depends on data quantity and quality. To address this, researchers have introduced advanced data mining techniques [10]. Ding Yuzi et al. [11] developed a machine learning algorithm for gas sensor arrays that effectively downscales and classifies sensor data, enhancing learning efficiency. Yu Cong et al. [12] created a BP neural network that accurately predicts toxic gas concentrations. Wang et al. [13] proposed an algorithm integrating long- and short-term attention with multi-task learning for the rapid, 30 s detection of gasses like CO and methane. Zhang et al. [14] designed a deep learning model (WCCNN-BiLSTM) for high-accuracy gas classification, achieving near-100% accuracy with fewer labels. Despite these advances, limited data hampers model generalization, prompting further research to boost accuracy under small-sample conditions. Wu et al. [15] addressed this with a TETCN-based classification method, employing Bayesian optimization, data weighting, and key feature extraction, surpassing traditional models like CNNs and LSTMs in terms of classification accuracy. Although the algorithms perform well in their respective fields, they often assume access to large, balanced datasets and lack adaptability to scenarios involving limited data. In contrast to prior studies using BP neural networks for toxic gas prediction, conventional TETCN-based classification or deep learning models like WCCNN-BiLSTM for high accuracy with labeled data, our approach uniquely addresses small, imbalanced datasets. This method preserves feature relationships and ensures robust classification, balancing efficiency and accuracy.

In machine learning model development, data quality and quantity are critical to model performance. Real-world scenarios often involve limited or imbalanced data, posing challenges to model accuracy and reliability. This imbalance leads to datasets where certain categories have far fewer samples than others, causing models to favor common categories and struggle with rare condition predictions. Such imbalance affects model bias, generalization, and metric skewness. With limited samples for minority categories, models may inadequately capture relevant features, reducing predictive accuracy in real-world applications. Decision rules learned on imbalanced data often skew towards majority categories, limiting generalization and weakening predictions for minority categories, while metrics like accuracy can misleadingly suggest high performance by overemphasizing majority predictions.

**Figure 1:**
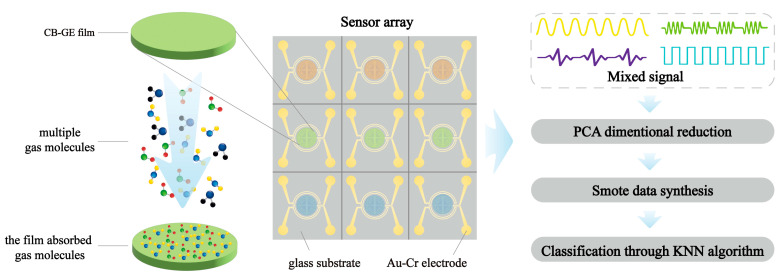
Flowchart of gas sensor data acquisition and classification process.

To address these challenges, this paper presents an optimized approach for finite dataset processing as shown in Figure 1, including feature extraction, polynomial fitting, and SMOTE-based synthesis. By integrating these techniques with multiple classification methods, we compare classification accuracy, analyze performance metrics, and demonstrate a promising application potential in mitigating data insufficiency and sensitivity limitations in specialized fields.

## 2. Methods

### 2.1. Principal Component Analysis

Principal Component Analysis (PCA) is a powerful technique for dimensionality reduction, enabling the effective processing of high-dimensional data [16,17]. Before applying PCA, data from different dimensions may exhibit correlations that can obscure analysis. PCA serves as an effective tool for extracting the primary features of the data.

**Figure 2:**
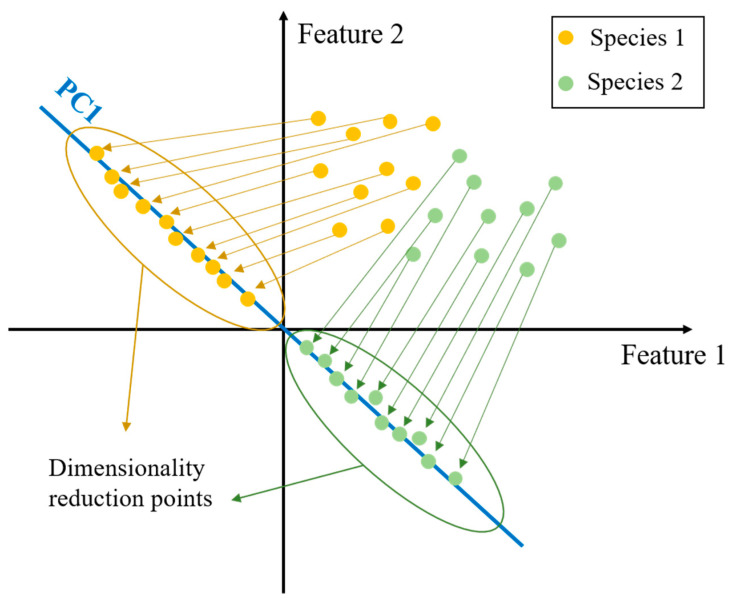
Schematic of the PCA algorithm for downscaling 2D data to one bit data.

For example, consider a scenario with two samples, “Species 1” and “Species 2”, each characterized by 2D feature data (“Feature 1” and “Feature 2”). The planar distribution of these features is illustrated in Figure 2. To extract the main information, PCA reduces the original 2D data to 1D by finding an axis similar to PC1 and projecting the 2D points onto this axis. The coordinates of the projected points represent the principal components, which capture the essential features of the data. The initial density of the sample points becomes more dispersed along the selected axis after dimensionality reduction.

### 2.2. Data Synthesis

Data generation refers to the generation of new data based on existing data or models, mainly based on statistical distributions, machine learning models, data augmentation, interpolation of data, and out-stacking. Data generation plays an important role in problem solving and is widely used to solve the problem of data scarcity or difficulty in collecting key data, data diversity to meet statistical needs, and security protection for sensitive data.

#### 2.2.1. Polynomial Fitting

**Figure 3:**
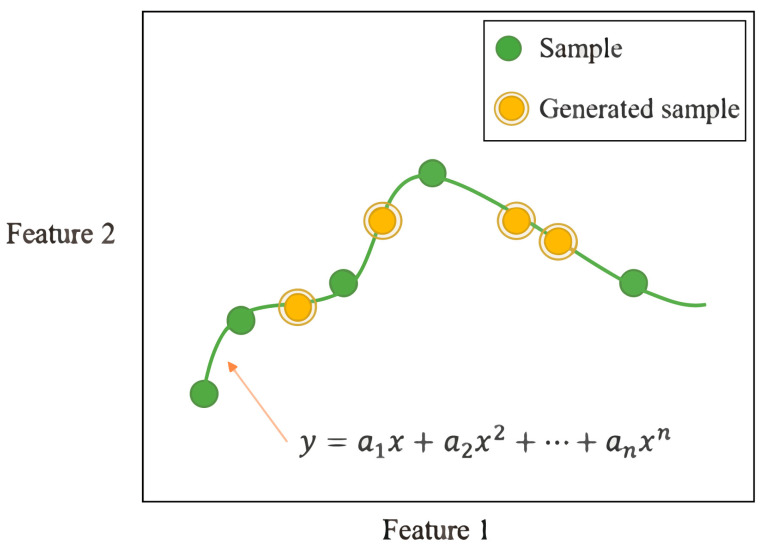
Polynomial you-sum principle and fitting generating value curve relationship based on sample data.

Polynomial fitting generates sample points by expanding a polynomial to match the observed data within a defined analysis region. This method finds a polynomial that aligns with the sample distribution, producing random values as new synthesized data points. The principle of polynomial fitting is illustrated in Figure 3.

#### 2.2.2. Smote Algorithm

**Figure 4:**
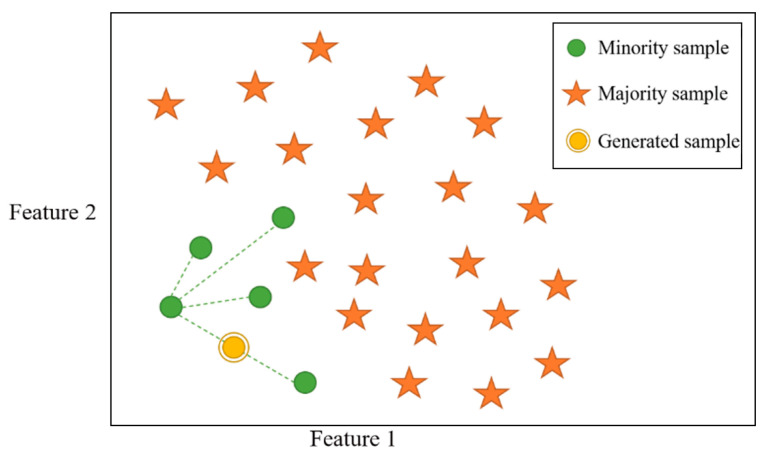
Schematic diagram of the generating value fitting principle based on the SMOTE algorithm.

The Synthetic Minority Over-sampling Technique (SMOTE) generates synthetic samples for minority classes by interpolating between existing data points, as illustrated in Figure 4. This method balances the dataset and enhances model performance on imbalanced data [18,19]. For gas sensor data, SMOTE preserves feature relationships while addressing data scarcity, thereby improving classification accuracy. By applying SMOTE, we generated synthetic data points that closely resemble the distribution of the original dataset, ensuring realistic augmentation without distorting feature correlations.

### 2.3. Data Classification

#### 2.3.1. Support Vector Machine Algorithm

Support Vector Machine (SVM), initially proposed by Vladimir Vapnik et al. [20], was developed for binary classification and later extended to multi-class classification and regression tasks. The core idea of SVM is to identify an optimal hyperplane in the feature space that separates different classes while maximizing the margin between them [21]. This hyperplane is defined by support vectors, the closest points to the hyperplane, allowing the model to classify new data mapped into the same space. SVM can handle both linear and nonlinear classification [22]. For linear classification, the goal is to find the hyperplane with the maximum margin, or the largest minimum distance between data points on either side, defining the maximum margin classifier. For nonlinear classification, kernel functions transform the feature space, enabling the algorithm to fit a maximum margin hyperplane. This paper focuses on linear classification.

For the category process, it has to do with the following functions. About the feature vector $x_{i}(i=1,2,\ldots ,N)$, the dataset’s label is $y_{i}\in \left\{\left.-1,1\right\}\right.$. The sample space is represented as follows:

(1) $$T=(x_{1},y_{1}),(x_{2},y_{2}),\ldots ,(x_{N},y_{N}),$$

Therefore, for a sample point, it is represented as $\left(x_{i},y_{i}\right)$. And the hyperplane is represented in the following part:

(2) $$\omega^{T}\cdot x+b=0,$$

(3) $$d=\frac{\left|\omega^{T}\cdot x+b\right|}{∥\omega ∥},$$

(4) $$\left\{\begin{array}{c} \omega^{T}\cdot x_{i}+b\geq 1,y_{i}=+1\\ \omega^{T}\cdot x_{i}+b\leq 1,y_{i}=-1 \end{array}\right.,$$

Within the expression, ω is the normal vector of the hyperplane, and b represents the constant, which defines the distance between the sample point and boundary. And d represents the distance between the sample point and the hyperplane. ∥ω∥ represents the magnitude of the normal vector. For example, when the sample point is put into the function and satisfies $\omega^{T}\cdot x+b>1$, this sample point belongs to $y_{i}=1$. On the contrary, if $\omega^{T}\cdot x+b<-1$, this sample point belongs to the category of $y_{i}=-1$.

As for the mathematical analysis, it has to do with the optimization problem. In the plot of the Support Vector Machine, the vectors on the boundaries are called support vectors. And the distance between those two boundaries is called the margin. The magnitude of the margin is shown in the following function. For the mathematical process, the optimization problem for seeking the largest margin equals to find the minimum of another target function in the following function:

(5) $$d=\frac{2}{∥\omega ∥},$$

(6) $$\underset{\omega ,b}{\max}\;\frac{2}{∥\omega ∥},$$

(7) $$\underset{\omega ,b}{\max}\;\frac{1}{2}∥\omega {∥}^{2},$$

#### 2.3.2. K-Nearest Neighbor Algorithm (KNN)

The K-Nearest Neighbor (KNN) algorithm, a supervised classification and regression method introduced by Evelyn Fix and Joseph Hodges in 1951 [23], classifies test samples by finding the nearest training samples and using majority voting to determine the test sample’s class. KNN calculates sample similarity using distance metrics like Euclidean, Manhattan, and Minkowski distances. For an unlabeled sample, KNN identifies the K nearest samples in the training set and assigns the class based on majority voting among these neighbors. The parameter K defines “proximity” and impacts classification accuracy and generalization. Typically, proximity is measured by Euclidean distance, defined as follows:

(8) $$L={\left(\sum_{n=1}^{n}|{x_{i}}^{n}-{x_{j}}^{n}{|}^{p}\right)}^{\frac{1}{p}},$$

where ${x_{i}}^{n}$ and ${x_{j}}^{n}$ are vector representations of two sample points. When p=2, $L=\sqrt{\sum_{n=1}^{n}{\left({x_{i}}^{n}-{x_{j}}^{n}\right)}^{2}}$, L is the Euclidean distance between the two sample points. When p=1, L is called the Manhattan distance between the two sample points. We can choose different *p* values according to the classification needs.

In general, KNN is a nonparametric, lazy-learning algorithm. It makes no assumptions about the sample data distribution (nonparametric) and does not involve an explicit training process (lazy-learning). This simplicity allows for quick setup and efficient processing. However, with a small dataset, KNN may struggle to classify effectively due to the limited training data available for comparison.

## 3. Experiments and Results

### 3.1. Preprocessing Stage

This study employs a special gas sensor array dataset created by Andrey Ziyatdinov as a sample [24]. We included Python software (version 3.12.1; Python Software Foundation, Wilmington, DE, USA) for data processing, algorithm execution, and image generation. Measurements were obtained using a chemical sensing system comprising an array of 16 metal oxide gas sensors and an external mechanical respirator simulating a biological respiratory cycle. The analytes—acetone and ethanol—were tested in 12 different mixtures or as pure gasses at various concentrations as shown in Table 1. Sensor output data, recorded as resistance values, were analyzed through data preprocessing, feature extraction, Principal Component Analysis (PCA), and comparative experiments to assess performance metrics.

Preprocessing gas sensor output signals is essential for building multi-target gas classification models and applying pattern recognition methods. Pattern recognition relies on the multi-dimensional features of each target, so it is crucial to standardize each dimension to ensure that data from different channels are comparable [25]. Additionally, the raw response from the sensor is typically weak and often contains significant noise. To prevent distortion in the pattern recognition results, it is essential to minimize noise through preprocessing. Thus, data preprocessing ensures a clean and standardized dataset, ready for the reliable application of pattern recognition methods.

#### 3.1.1. Normalization of Data

The gas sensor array dataset is processed by the normalized method. The value stored in the dataset is the corresponding load resistance value in the experiment. The data point stored in the original dataset is the resistance value R(t) of the sensor at a specific time t. After that, the original data are preprocessed. We subtract the initial baseline resistance R(0) from the resistance at a specific point in time and then scale the results according to the baseline resistance as a scaling factor. Finally, a new dataset for implementing the subsequent machine learning algorithm is obtained. The normalization process is summarized as the following formula:

(9) $$R_{new}=\frac{R(t)-R(0)}{R(0)},$$

where $R_{new}$ is the normalized resistance value and R(t) and R(0) are the resistance value at specific time t and the initial baseline resistance value, respectively.

#### 3.1.2. The Filtering Processing of Data

**Figure 5:**
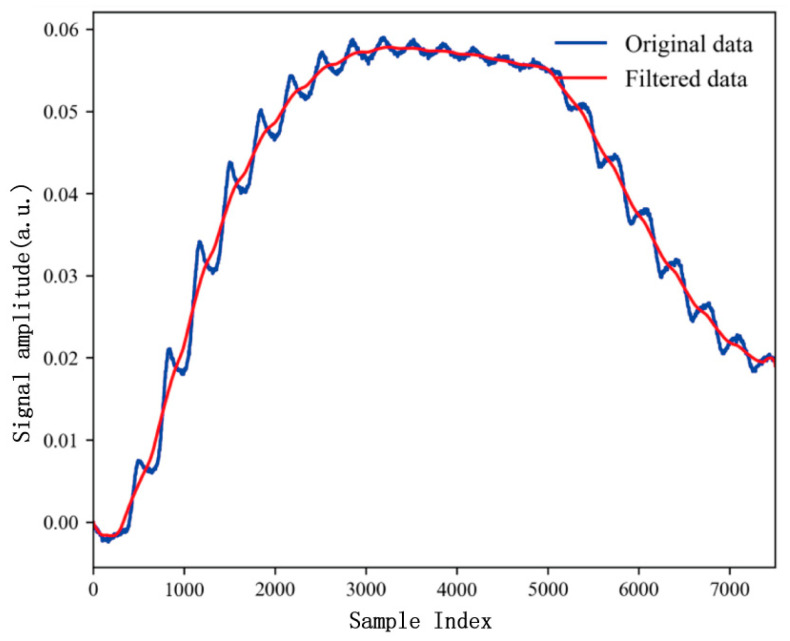
Raw data curves and data fitting curves for the filtering results of the moving average filter.

Gas signals often contain noise and extraneous data, which can lead to errors in target gas identification. To address this, we can apply numerical operations, such as moving average filters, to remove irrelevant information. This approach effectively smooths the raw data curve by filtering out “spike” data. The window size for the moving average filter was selected based on the characteristics of the gas sensor signals, such as signal fluctuation frequency and noise level. A window length of three was chosen to ensure that the filter effectively smooths the data without excessively dampening critical variations. The original and filtered data are shown in Figure 5, where “original data” represent the unprocessed signal collected by the gas sensor, and “new data” are the signal after applying moving average filtering. This filtering technique successfully smooths the data. Nevertheless, it can also lead to a loss of signal resolution, particularly for sharp transitions or high-frequency components. This trade-off should be considered when applying the filter to gas sensor data, as excessive smoothing may obscure subtle but critical variations in the signal that are important for classification accuracy. However, in our case, the moving average filter has a more positive effect by significantly enhancing signal clarity without compromising critical information for classification.

#### 3.1.3. Butterworth Filters of Third Order

After using a median filter to remove the spikes in the signals, we employed two Butterworth filters of the third order. The Butterworth filter is a typical analog filter [26]. Compared with other types of analog filters, the Butterworth filter has the advantage of “flatter”, that is, in the passband range, the frequency response is more stable, and the fluctuation is small. The transfer function can be determined as follows:

(10) $$|A(\omega ){|}^{2}=\frac{1}{1+{\left(\omega /\omega_{c}\right)}^{2n}},$$

where A(ω) is the amplitude–frequency characteristics of the filter, ω is the frequency, $\omega_{c}$ is the transition frequency, and n is the filter order.

**Figure 6:**
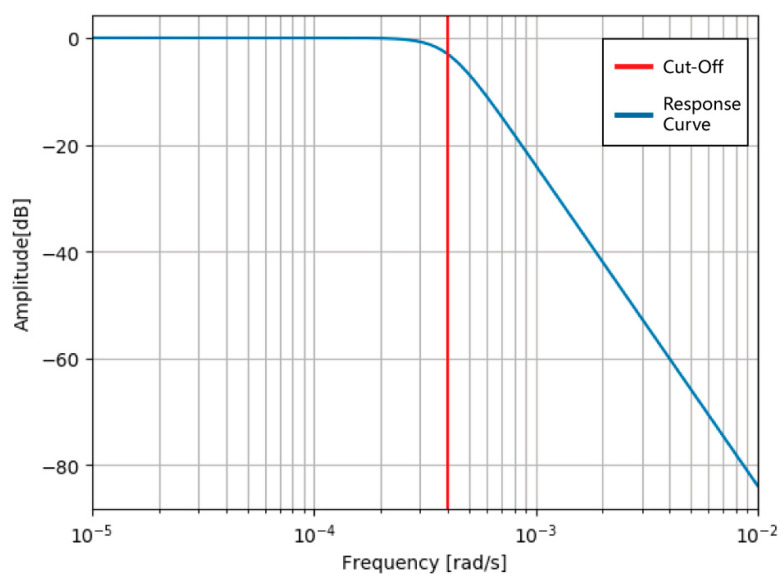
Frequency response curve of a third-order Butterworth filter.

The frequency response curve of the Butterworth filter in the stopband shows a gradual decrease in the attenuation rate with increasing order [27]. The third order Butterworth filter is built using Python, and its normalized cutoff frequency is calculated as follows:

(11) $$\omega_{n}=\frac{2\times f_{c}}{f_{s}},$$

where $\omega_{n}$ is the normalized cutoff frequency, $f_{c}$ is the cutoff frequency, and $f_{s}$ is the sampling frequency. According to the Nyquist sampling theorem, the sampling frequency must be greater than two times the maximum frequency of the original signal to achieve the restoration of the original signal. The cutoff frequency of the low-pass filter is 0.01; therefore, the normalized cutoff frequency is $\omega_{n}=\frac{2*0.01}{2*25}=0.0004$. Then, the corresponding frequency response is calculated, and the results are shown in Figure 6.

This filter exhibits an attenuation rate of 60 dB per decade, consistent with a third-order filter. In the figure, the red vertical line indicates the normalized cutoff frequency, marking the threshold where significant attenuation begins. Below this frequency, the signal is well preserved, while above it, rapid attenuation occurs. This demonstrates the effectiveness of the third-order Butterworth filter in providing a smooth frequency response within the passband while sharply attenuating unwanted high-frequency signals in the stopband, thereby ensuring signal stability and effective filtration.

### 3.2. Feature Extraction

**Figure 7:**
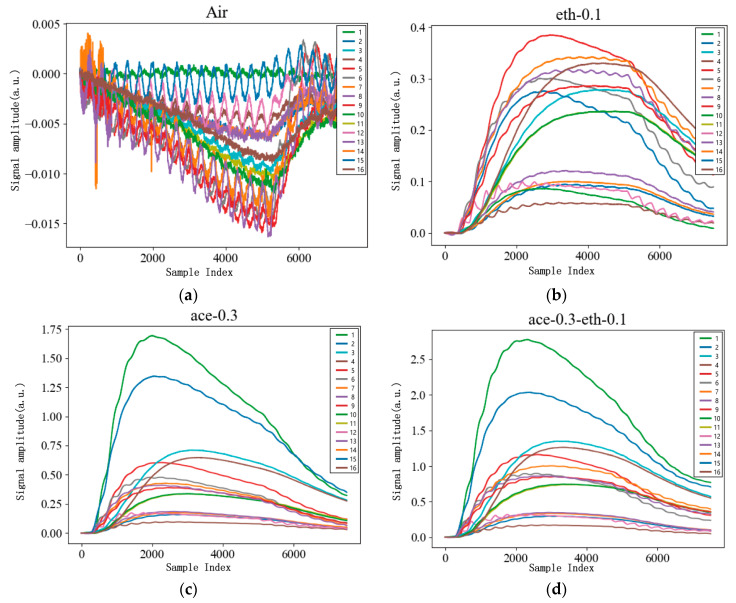
Plot of sensor responses to different gas mixtures: (**a**) response curve of 16 sensor outputs in air; (**b**) response curve of 16 sensor outputs in eth-0.1; (**c**) response curve of 16 sensor outputs in ace-0.3; (**d**) response curve of 16 sensor outputs in ace-0.3-eth-0.1.

In the measurement system, signals are recorded at a sampling frequency of 25 Hz over a 5 min period, generating 7500 data points per time series for each sensor. Four types of gasses are randomly selected and visualized using computational tools, with each sensor’s response to different gas mixtures displayed in Figure 7.

**Figure 8:**
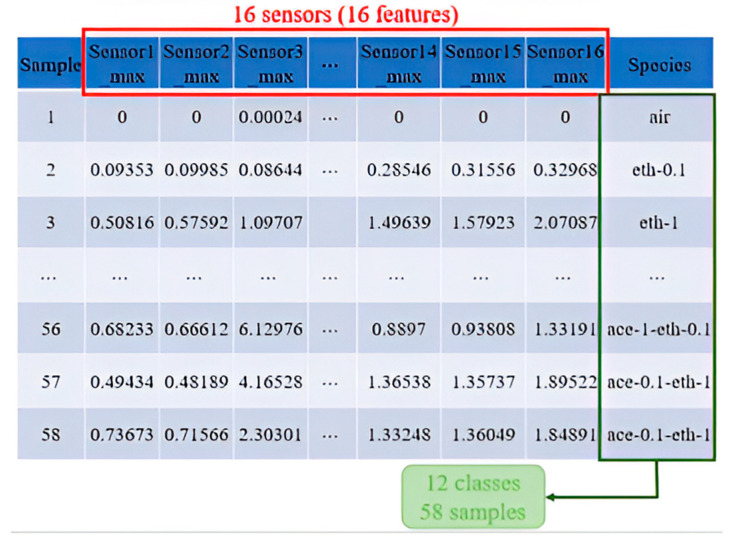
A total of 16 gas sensor signal datasets for 58 samples.

Two Butterworth filters of the third order are used: a low-pass filter (cutoff frequency 0.01 Hz) and a high-pass filter (pass-frequency 0.07 Hz) to generate low- and high-frequency signals [28]. After the process of the low-pass filter, it is found that there is one maximum value for each sample. After the process of the high-pass filter, it is found that the signal shapes like a sine wave, so that each cycle can be used to extract features like trough or crest. In this experiment, the maximum value of each feature of each sample is used as the feature for each gas sensor, as shown in Figure 8.

### 3.3. Feature Reduction (PCA)

In this experiment, 58 samples are analyzed, with each sample containing 16 features extracted via the low-pass filter. During PCA processing, these 16-dimensional features are reduced to two principal components for classification.

**Figure 9:**
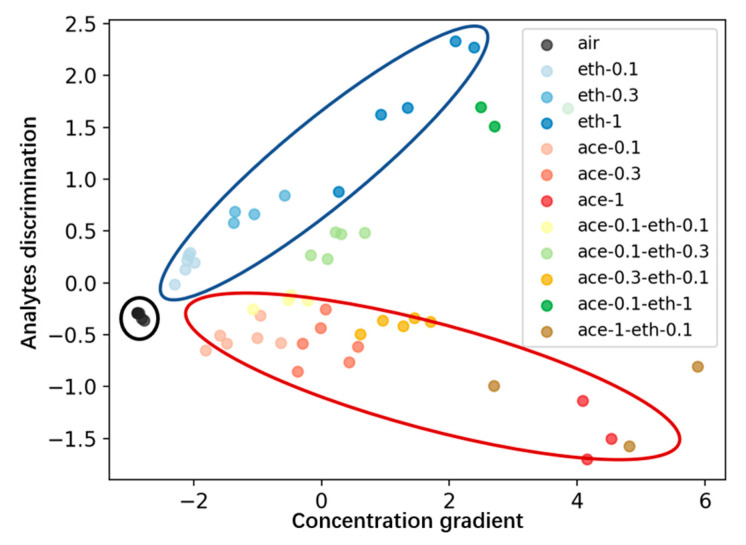
Plotting of data from 16 sensors after processing by PCA algorithm.

In Figure 9, each point represents one of the 58 samples. The *x*-axis corresponds to the new Feature I, while the *y*-axis represents the new Feature II. The data points are distinctly grouped into four categories: air samples on the left, ethanol samples in the top-left, acetone samples in the bottom-right, and mixture samples in the center. The *x*-axis reflects the increasing concentration, while the *y*-axis indicates the analyte resolution.

### 3.4. Data Synthesis

#### 3.4.1. Polynomial Fitting

In this study, the dataset exhibits significant category imbalances, which can substantially affect the classification accuracy. The original dataset includes only 58 samples across 12 categories, with each category containing insufficient samples for robust data generation. Initially, we attempted to augment the dataset using a polynomial fitting method. Although the data distribution plot does not show a clear linear distribution within each category, nearly all categories form distinct clusters. Therefore, we chose to fit a polynomial to the data for categories with fewer samples, selecting an optimal polynomial order based on the preliminary results. Random values within suitable intervals were then substituted into the polynomial, producing new synthetic sample data. These random inputs, combined with the corresponding polynomial outputs, serve as newly generated sample points. The polynomial fitting method, based on actual measured data points for the target gasses, provides a fitted curve as a function of

(12) $$y=-8.624\times 10^{9}x^{5}+5.931\times 10^{6}x^{4}+6.672\times 10^{5}x^{3}-1935x^{2}+2.135x-2.208\times 10^{-17},$$

**Figure 10:**
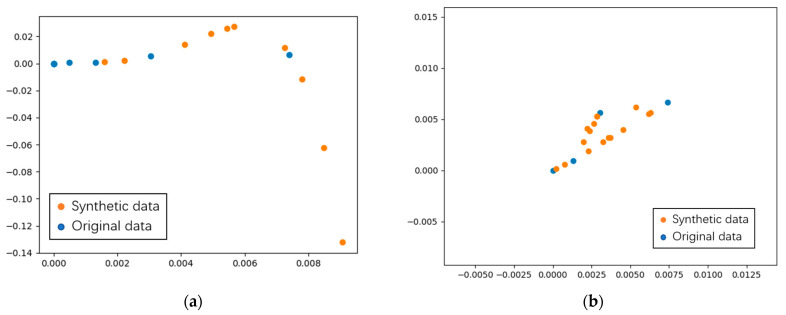
Data fitting results using (**a**) polynomial fitting and (**b**) SMOTE algorithm.

The results of the data synthesis are presented in Figure 10a, where the orange points represent the original sample data and the blue points indicate the newly generated samples created through polynomial fitting.

As illustrated in Figure 10a, the generated points align closely with the original sample points on the same curve, highlighting a limitation of this approach. This method risks distorting the original data’s distribution characteristics. If the original points lack an inherent “linear” relationship, this technique may inadvertently impose a misleading high degree of linear correlation among the points.

#### 3.4.2. Application of SMOTE Algorithm

Alternatively, the SMOTE algorithm can be applied to augment artificial samples. For this experiment, we randomly selected four sample points and used the SMOTE algorithm to generate additional data. The results are displayed in Figure 10b, where the blue points represent the original samples, and the orange points indicate the synthetic samples. As observed in the figure, the SMOTE algorithm preserves the relationships between the original features, avoiding distortion of the data’s inherent structure.

### 3.5. Data Classification

Data classification is a fundamental task in machine learning and data mining, aiming to categorize data into predefined classes or labels based on specific characteristics. This technique has broad applications, including image recognition, text categorization, predictive analytics, and recommendation systems. Initially developed within the realms of statistics and pattern recognition, classification methods have evolved significantly with advances in machine learning algorithms, establishing themselves as essential tools for addressing real-world problems.

#### 3.5.1. Application of Support Vector Machine

As noted, Support Vector Machines (SVMs) are effective for classifying small datasets. Combining Principal Component Analysis (PCA) with SVMs can enhance classification accuracy [29]. The following figure illustrates the classification outcomes after integrating PCA and SVM algorithms, with gas types labeled from 0 to 11. Structurally, the program is organized into two main components: the PCA and SVM models. The PCA model processes 12 original features to extract 2 new features for further analysis.

**Figure 11:**
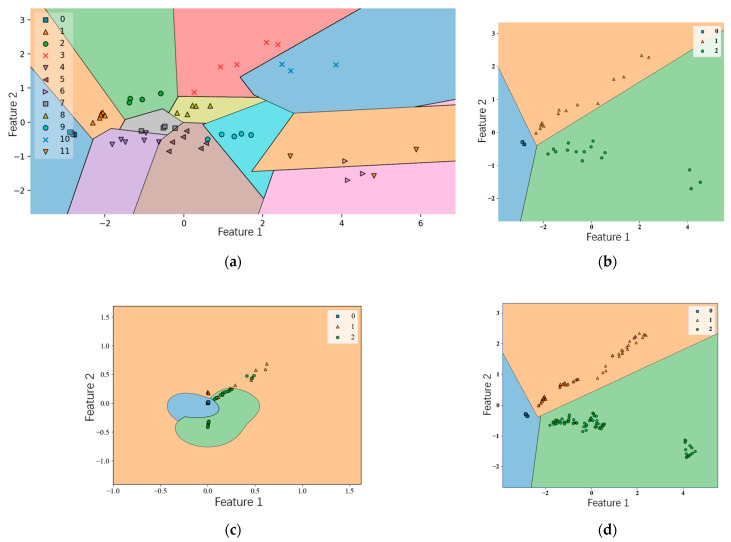
Classification results of support vector machine at different stages of data processing. (**a**) Initial classification using 12 features; (**b**) simplified classification with three merged categories; (**c**) nonlinear boundary classification using kernel technique; (**d**) classification after data augmentation with SMOTE.

In the SVM model, the first feature is plotted along the *x*-axis and the second along the *y*-axis [30]. In the initial classification phase, the mixture is categorized into 12 gas types. Using the SVM, the entire dataset is segmented into 12 distinct, color-coded regions, as clearly depicted in Figure 11a.

However, a notable drawback of the initial approach is the excessive number of regions, resulting in a cluttered and less interpretable graph. To address this, the gas types were further consolidated into three main categories: pure ethanol, pure acetone, and mixtures of ethanol and acetone. This refinement produces a more straightforward and interpretable graph, as shown in Figure 11b, where the data are segmented into clear sections for each of these three types. According to the program results, this improved classification yields an accuracy of 91.7%.

In the previous section, given the limited sample size for each gas category, an artificial synthesis approach is necessary. Assuming a “linear” distribution of the data points, we applied a polynomial fitting method to generate additional sample data, followed by Support Vector Machine (SVM) classification of the expanded dataset [31]. Since the dataset becomes “nonlinearly separable” after synthetic data generation, linear classification boundaries are no longer suitable. Consequently, we employed the SVM kernel technique to establish nonlinear boundaries. The classification results, shown in Figure 11c, depict the boundaries dividing the three target gasses into distinct regions, effectively separating each gas type.

This approach, assuming a temporarily linear data distribution, utilizes polynomial fitting for data generation, followed by SVM classification with kernel techniques to define nonlinear boundaries for the expanded dataset [32].

The SMOTE algorithm synthesizes new samples based on the distance between existing samples, preserving the correlation between features and effectively addressing the issue of limited data points. Therefore, SMOTE is a suitable choice for sample data synthesis. The classification results for the SMOTE-augmented dataset, processed by a Support Vector Machine (SVM), are shown in Figure 11d. As shown in the figure, while the total number of samples remains relatively limited, the feature distribution of each gas type is distinguishable. For air, the measured signals are minimal, nearly all clustering around zero, resulting in overlapping sample points. Acetone sample points exhibit an approximately linear distribution, whereas ethanol sample points are more concentrated, forming a distinct cluster. Overall, the SMOTE algorithm effectively synthesizes data and expands small sample sets, enhancing the dataset’s representativeness.

#### 3.5.2. Application of KNN Algorithm

**Figure 12:**
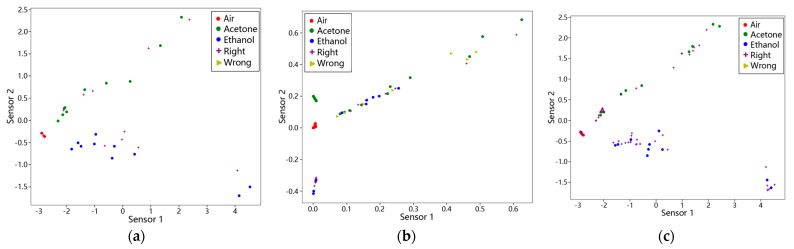
Classification results of KNN algorithm under different data processing methods. (**a**) KNN classification results after PCA dimensionality reduction; (**b**) classification results of KNN on polynomially fitted synthetic data; (**c**) classification results of KNN on SMOTE-synthesized data.

As previously discussed, the K-Nearest Neighbor (KNN) algorithm is a simple yet effective classification method. In this analysis, we categorized the dataset into three classes—air, acetone, and ethanol—and trained the model using data points calculated through Principal Component Analysis (PCA). The classification results are shown in Figure 12a, where purple plus signs indicate correct classifications and yellow triangles denote errors. The KNN algorithm achieved nearly 100% accuracy in distinguishing the three gas types, indirectly validating the effectiveness of PCA [33]. The three gasses display distinct distribution patterns: acetone aligns linearly at the top, air clusters around a single point, and ethanol appears at the bottom.

We also applied the KNN algorithm to classify synthetic data generated by polynomial fitting. Figure 12b shows the classification results, with purple plus signs for correct points and yellow triangles for errors. The polynomial fitting produced data that were too dispersed, resulting in lower classification accuracy and increased errors.

In contrast, SMOTE, as mentioned earlier, preserves the relationships among original features. When KNN was used to classify SMOTE-synthesized data, as shown in Figure 12c, the prediction accuracy was close to 100%, with significantly fewer errors. Overall, SMOTE’s synthesis approach aligns effectively with KNN classification, yielding a more accurate prediction model.

### 3.6. Results and Discussion

The classification results of the proposed technique were evaluated using performance metrics and compared with those from classifications conducted without synthetic data and without PCA. This paper employs the Receiver Operating Characteristic (ROC) curve and the Area Under the Curve (AUC) value to assess the model’s generalization capability [34,35,36]. In classification, a True Positive TP occurs when the model correctly identifies a positive instance, while a False Negative FN occurs when the model misclassifies a positive instance as negative. Conversely, a False Positive FP is when the model incorrectly classifies a negative instance as positive, and a True Negative TN is when it correctly identifies a negative instance.

From these classifications, two key metrics are derived:

(13) $$TPR=\frac{TP}{TP+FN},$$

The True Positive Rate TPR represents the likelihood of correctly identifying positive cases among actual positives.

(14) $$FPR=\frac{FP}{FP+TN},$$

The False Positive Rate FPR indicates the probability of misclassifying negative cases as positive.

In binary classification, a threshold determines whether a prediction is positive or negative. Points on the ROC curve represent different threshold values, with FPR on the horizontal axis and TPR on the vertical axis. The AUC value measures the area under the ROC curve, providing a single metric for model performance across thresholds.

#### 3.6.1. Comparison of Classification with and Without Data Processing Techniques

**Figure 13:**
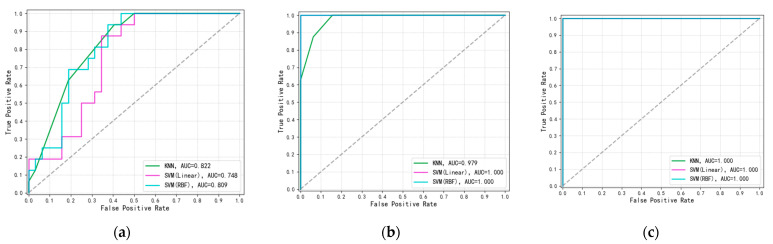
ROC curves and AUC values of classifiers with different data processing techniques. (**a**) Classifier performance before PCA dimensionality reduction; (**b**) classifier performance after PCA dimensionality reduction (before SMOTE data synthesis); (**c**) classifier performance after SMOTE data synthesis.

The performance of the proposed methods was evaluated using Receiver Operating Characteristic (ROC) curves and the Area Under the Curve (AUC) metric, as shown in Figure 13. These metrics were chosen because they effectively measure the classifiers’ ability to distinguish between classes across various decision thresholds.

We first assessed the classification performance of the dataset before applying PCA for dimension reduction, as shown in Figure 13a. Two classifiers, SVM and KNN, were used for this evaluation, with SVM employing both linear and RBF kernel functions. According to the figure, KNN shows slightly better classification performance than SVM on the dataset prior to PCA.

Next, we evaluated the dataset’s classification performance after PCA dimension reduction, as depicted in Figure 13b. Post-PCA, both classifiers achieved near-perfect performance, underscoring the effectiveness of PCA in extracting the primary features of the data.

As noted earlier, the distribution of points generated through polynomial fitting significantly differs from that of the original sample points. While the original data points exhibit a roughly linear distribution, the generated points are more dispersed. This illustrates the main drawback of polynomial fitting: synthesizing a large number of samples with this method can easily distort the original feature distribution. Since the classification model is intended to represent the majority of the sample data, altering the original feature distribution undermines the model’s relevance. In contrast, as shown in Figure 13c, SMOTE preserves feature correlations and effectively addresses the issue of limited dataset size, making it a more suitable choice. Consequently, we ultimately chose SMOTE for data augmentation.

Table 2 presents the comparison of AUC values for KNN and SVM classifiers under different data processing techniques, highlighting the performance improvements achieved through PCA and SMOTE. For the original dataset, the AUC values for KNN, SVM (Linear), and SVM (RBF) were 0.822, 0.748, and 0.809, respectively, reflecting the limitations of the unprocessed data. After applying PCA, the AUC value of KNN increased by 0.157 to 0.979, indicating that PCA effectively extracted key features and improved generalization. Further enhancement with SMOTE brought all classifiers to an AUC of 1.000, demonstrating the effectiveness of SMOTE in balancing the dataset and preserving feature relationships. Overall, while SVM achieved perfect classification after PCA and SMOTE, KNN showed slightly better adaptability, with higher initial improvement from the original dataset.

#### 3.6.2. Classification Results

According to the experimental results with the Support Vector Machine (SVM) and KNN algorithms, the polynomial fitting method may compromise the original data characteristics due to its linearity constraints. In contrast, the SMOTE algorithm generates new data points based on the distance between gas samples, using random sampling that preserves the original characteristic relationships within the data.

The evaluation of model generalization reveals that PCA effectively enhances classification performance by isolating key features, while SMOTE sharpens the definition of the classification boundaries. As illustrated in Figure 13, removing either component (PCA or SMOTE) results in a noticeable drop in model accuracy, highlighting their importance.

A detailed analysis is as follows:

- Impact of Removing PCA: After removing PCA, the AUC for KNN decreased by 0.157, while SVM’s AUC declined by 0.252 with a linear kernel and 0.191 with an RBF kernel. This indicates that PCA significantly enhances classification accuracy, with KNN experiencing a smaller accuracy reduction than SVM, supporting the hypothesis that KNN combined with SMOTE yields superior results.
- Impact of Removing SMOTE: When the SMOTE synthesis mechanism is removed, the KNN classification accuracy drops by 0.021. This demonstrates that SMOTE aids the classification model by emphasizing data characteristics, ultimately enhancing model performance.

## 4. Conclusions

This paper introduces a data synthesis and classification model for gas sensor data. A dataset of 58 samples containing 12 gas types (mixtures and pure gasses) across 16 features, collected through a metal oxide gas sensor array, forms the basis for testing the model. The framework of the model is incrementally developed, with components tested and refined to optimize classification performance, demonstrating its effectiveness for small gas sensor datasets.

1. Combining PCA and SVM, gas samples are classified into three types—pure ethanol, pure acetone, and ethanol–acetone mixtures—achieving a classification accuracy of 91.7%.
2. Polynomial fitting is used to generate additional sample points, but the dispersed distribution affects classification accuracy. SMOTE, however, effectively expands the dataset without disrupting the feature distribution.
3. PCA and KNN algorithms applied to classify air, acetone, and ethanol samples yield nearly 100% accuracy, confirming PCA’s effectiveness.
4. SMOTE maintains original feature relationships, addressing sample size limitations. KNN achieves nearly 100% prediction accuracy with SMOTE-synthesized data.
5. The model’s generalization is evaluated using ROC and AUC metrics. Before PCA, KNN slightly outperforms SVM; after PCA, both classifiers approach perfect classification, which further improves with SMOTE.
6. PCA significantly enhances classification performance, with KNN AUC increasing by 15.7% and SVM by 25.2% and 19.1%. KNN with SMOTE outperforms other configurations. Removing SMOTE reduces KNN’s accuracy by 2.1%, underscoring SMOTE’s role in refining classification boundaries based on data features.

## Figures and Tables

**Table 1:** Gas properties of each experimental group.

Pure Gas	Gas Mixture
eth-0.1	ace-0.1-eth-0.1
eth-0.3	ace-0.1-eth-0.3
eth-1	ace-0.3-eth-0.1
ace-0.1	ace-0.1-eth-1
ace-0.3	ace-1-eth-0.1
ace-1	air

**Table 2:** Comparison of AUC value of classifiers with different processing techniques.

Classifiers	Original	After PCA	After SMOTE
KNN	0.822	0.979	1.000
SVM (Linear)	0.748	1.000	1.000
SVM (RBF)	0.809	1.000	1.000

## Data Availability

The original contributions presented in this study are included in the article. Further inquiries can be directed to the corresponding authors.
